# Diagnostic accuracy of ultrasonography for lower extremity arterial disease in patients with diabetes

**DOI:** 10.1097/MD.0000000000048885

**Published:** 2026-05-15

**Authors:** Junxiang Zhao, Huanping Zhang, Qian Zhang, Ling Sun, Jingjing Kong, Jie Gao, Chunyan Shao

**Affiliations:** a Ultrasound Department, Cangzhou Hospital of Integrated Traditional Chinese and Western Medicine, Cangzhou City, Hebei Province, China; b Internal Medicine Department, Renqiu People’s Hospital, Renqiu City, Hebei Province, China.

**Keywords:** computed tomography angiography, diabetes mellitus, diagnostic accuracy, duplex ultrasonography, lower extremity arterial disease

## Abstract

Lower extremity arterial disease (LEAD) is a major macrovascular complication in patients with diabetes mellitus, and accurate noninvasive imaging is essential for early recognition and risk stratification. This retrospective diagnostic accuracy study included 59 adult patients with diabetes who underwent both lower extremity arterial ultrasonography and contrast-enhanced computed tomography angiography (CTA) within a predefined interval of ≤ 30 days. Baseline data showed that patients with CTA-confirmed LEAD were older, had longer diabetes duration, higher glycated hemoglobin (HbA1c), a higher prevalence of hypertension, lower estimated glomerular filtration rate (eGFR), and higher low-density lipoprotein cholesterol (LDL-C) than those without LEAD. CTA revealed a substantial burden of disease with frequent multisegment involvement across iliac–femoral, popliteal, and infrapopliteal segments. Using CTA as the reference standard and a threshold of ≥ 50% diameter stenosis or occlusion, ultrasonography demonstrated a sensitivity of 90.0% and specificity of 75.9% at the patient level, and a sensitivity of 88.5% and specificity of 89.7% at the segment level. Negative predictive values were high at both levels, and agreement between ultrasonography and CTA was acceptable by McNemar’s test. Detection rates increased with lesion severity, with excellent performance for moderate and severe stenoses. These findings indicate that duplex ultrasonography provides robust diagnostic information on clinically significant LEAD in patients with diabetes and can be used as a reliable imaging modality in routine clinical practice.

## 1. Introduction

Lower extremity arterial disease (LEAD) is a frequent macrovascular complication of diabetes mellitus and contributes substantially to functional impairment, ulceration, limb loss, and excess cardiovascular mortality. Recent epidemiological studies indicate that people with type 2 diabetes have a markedly higher risk of peripheral artery disease (PAD) and lower-extremity revascularization or amputation than the general population, despite improvements in risk factor management over time.^[1]^ Contemporary cohorts of previously unscreened individuals with type 2 diabetes continue to show a notable proportion of asymptomatic PAD, underscoring the need for systematic vascular assessment in this group.^[2]^ The coexistence of advanced age, prolonged diabetes duration, suboptimal glycemic control, hypertension, dyslipidemia, and renal dysfunction further amplifies the risk of LEAD and its complications.^[3]^ LEAD in diabetes is often diffuse and multisegmented, with a predilection for distal intrahospital vessels, and is a key driver of diabetic foot ulceration and chronic limb-threatening ischemia.^[4]^ International Working Group on the Diabetic Foot (IWGDF) and intersocietal PAD guidelines emphasize early recognition of LEAD as a central component of limb-preserving care, advocating structured vascular examination and timely imaging to delineate disease severity and distribution.^[5]^ However, the optimal diagnostic pathway in patients with diabetes remains a subject of ongoing debate, particularly given the limitations of resting ankle–brachial index in the presence of medial arterial calcification and distal vessel involvement.^[6]^

Anatomic imaging modalities, including computed tomography angiography (CTA), magnetic resonance angiography, and digital subtraction angiography, provide detailed visualization of the arterial tree and are widely used for pre-interventional planning. CTA offers rapid, high-resolution depiction of luminal stenosis and occlusion, but its use is constrained by ionizing radiation, iodinated contrast, and reduced accuracy in heavily calcified segments.^[7]^ Duplex ultrasonography, in contrast, is noninvasive, contrast-free, and relatively inexpensive, and can provide both morphologic and hemodynamic information on lower extremity arteries. Recent work has explored advanced Doppler parameters and quantitative flow metrics for PAD detection, including contrast-free ultrasound-based blood flow imaging and refined spectral acceleration indices, suggesting that contemporary ultrasound techniques may further improve diagnostic performance, particularly in patients prone to medial calcification.^[8]^

Despite these developments, data specifically addressing the diagnostic accuracy of lower extremity ultrasonography in patients with diabetes, using CTA as the reference standard and with segment-wise and severity-stratified analyses, remain limited. Existing studies in diabetic cohorts have often focused on mixed imaging strategies or have not systematically quantified sensitivity, specificity, and predictive values at both patient and segment levels.^[9]^ Moreover, few investigations have explicitly examined performance in intrahospital segments, where diabetic atherosclerosis and calcification are particularly prevalent and imaging is technically challenging. In this context, a detailed evaluation of duplex ultrasonography against CTA in a well-characterized diabetic population is needed to clarify its role as a first-line structural imaging modality and to define its strengths and limitations across arterial territories and lesion severities.

## 2. Methods

### 2.1. Study design

This study was approved by the Ethics Committee of Cangzhou Hospital of Integrated Traditional Chinese and Western Medicine.This retrospective diagnostic accuracy study included adult patients with diabetes mellitus who underwent lower extremity arterial ultrasonography and clinically indicated lower extremity computed tomography angiography (CTA) between January 2023 and December 2024 at our institution. Eligible participants were individuals aged 18 years or older with a confirmed diagnosis of diabetes mellitus according to established clinical criteria, who received both ultrasonography and CTA within a predefined time interval and had complete clinical and imaging data available. Exclusion criteria included acute lower limb ischemia at presentation, known nonatherosclerotic arterial diseases such as vasculitis or thromboangiitis obliterans, previous lower extremity vascular surgery or endovascular intervention, severe renal insufficiency or documented allergy to iodinated contrast media precluding CTA, pregnancy or lactation, incomplete medical records, and ultrasonography or CTA images of insufficient quality to permit reliable interpretation. The study adhered to the principles of the Declaration of Helsinki and received approval from the institutional medical ethics committee. Written informed consent was obtained from all participants before inclusion. Patients were identified through a consecutive review of the institutional database. Eligible patients were identified through consecutive retrospective review of the institutional radiology and vascular laboratory databases between January 2023 and December 2024.

### 2.2. Data collection and imaging assessment

Clinical and imaging data for all eligible patients were obtained retrospectively from the institutional electronic medical record and picture archiving systems. For each patient, baseline demographic and clinical variables included in the analysis were age, sex, and body mass index (BMI). Diabetes-related information comprised duration of diabetes and the most recent glycated hemoglobin (HbA1c) value recorded before imaging. Vascular risk factors collected were the presence or absence of hypertension, dyslipidemia, coronary artery disease, and current smoking status. Renal function was characterized using the estimated glomerular filtration rate (eGFR), and lipid status was characterized using low-density lipoprotein cholesterol (LDL-C), as documented in the medical record.

Imaging data consisted of lower extremity arterial ultrasonography and contrast-enhanced computed tomography angiography (CTA). Only patients who underwent both examinations within a predefined interval were included to reduce the risk of interval change in arterial status. For each patient, the iliac–femoral, popliteal, and intrahospital arterial segments were evaluated bilaterally, yielding 3 predefined segments per limb. For both ultrasonography and CTA, the presence and degree of stenosis in each segment were recorded according to routine departmental practice and classified into 4 categories: no stenosis, mild stenosis (<50% diameter reduction), moderate stenosis (50–69% diameter reduction), and severe stenosis or occlusion (≥70% diameter reduction or complete occlusion).

CTA served as the reference standard for all diagnostic performance analyses. At the segment level, a “clinically significant” lesion was defined as ≥ 50% diameter stenosis or occlusion on CTA. At the patient level, clinically significant lower extremity arterial disease (LEAD) was defined as the presence of at least 1 arterial segment with ≥ 50% stenosis or occlusion on CTA. Corresponding ultrasonographic classifications were extracted using the same segment definitions and stenosis thresholds to allow direct patient level and segment-level comparisons between ultrasonography and CTA. All imaging assessments used in the analysis were derived from finalized clinical reports issued by experienced vascular sonographers and radiologists who were unaware of the aims of the present study. Ultrasonography was performed using a standardized protocol by 2 attending vascular sonographers (J.Z., H.Z., each with > 8 years of experience). Each examination was interpreted by a single sonographer who was blinded to the CTA findings, and the finalized clinical report was used for study analysis. CTA was interpreted by a single attending radiologist (C.S., 15 years of experience) without knowledge of the ultrasound results. Images were assessed independently, ensuring mutual blinding between modalities.

## 3. Statistical analysis

All statistical analyses were performed using IBM SPSS Statistics, version 26.0 (IBM Corp., Armonk, NY, USA). Continuous variables (e.g., age, BMI, duration of diabetes, HbA1c, eGFR, LDL-C) were summarized as mean ± standard deviation and compared between the LEAD and non-LEAD groups using independent-samples t-tests. Categorical variables (e.g., sex, hypertension, dyslipidemia, coronary artery disease, current smoking, presence of CTA-defined lower extremity arterial disease, and multisegmented involvement) were presented as counts and percentages and compared using the χ^2^ test. For diagnostic performance analyses, CTA served as the reference standard. At both the patient and segment levels, 2 × 2 contingency tables were constructed to derive sensitivity, specificity, positive predictive value (PPV), negative predictive value (NPV), and overall accuracy of ultrasonography for detecting clinically significant lesions (≥50% diameter stenosis or occlusion). Corresponding 95% confidence intervals (CIs) for these proportions were calculated using the binomial distribution. Agreement between ultrasonography and CTA classifications at the patient and segment levels was further evaluated using McNemar’s test for paired proportions. Differences in sensitivity and specificity among iliac–femoral, popliteal, and infrapopliteal segments were assessed using χ^2^ tests for comparison of proportions. All statistical tests were 2-sided, and a *P*-value < .05 was considered statistically significant. No a priori sample size calculation was performed due to the retrospective design. Instead, a post hoc power analysis was conducted for the primary endpoint (patient-level sensitivity). Using an observed sensitivity of 0.90, a null hypothesis sensitivity of 0.70, 30 disease-positive cases, and one-sided α = 0.05, the achieved power was 0.92 (PASS 15, NCSS). Statistical significance was set at *P* < .05 (two‑tailed). Analyses were performed with SPSS 26.0.

## 4. Results

### 4.1. Baseline demographic and clinical characteristics

A total of 59 patients with diabetes mellitus were included, comprising 36 patients in the LEAD group and 23 patients in the non-LEAD group. Baseline characteristics are summarized in Table 1. Patients in the LEAD group were older than those in the non-LEAD group (70.1 ± 7.7 vs 60.0 ± 7.7 years; t = 4.91, *P* < .001). Sex distribution did not differ significantly between groups (male: 61.1% vs 56.5%; χ^2^ = 0.12, *P* = .726). Body mass index (BMI) was comparable (24.8 ± 2.8 vs 25.5 ± 3.4 kg/m^2^; t = −0.82, *P* = .414). The duration of diabetes was significantly longer in the LEAD group (12.6 ± 4.6 vs 7.8 ± 3.9 years; t = 4.29, *P* < .001), and glycated hemoglobin (HbA1c) levels were higher (8.4 ± 1.2% vs 7.6 ± 0.8%; t = 3.07, *P* = .003), indicating poorer glycemic control. Hypertension was more prevalent in the LEAD group (75.0% vs 43.5%; χ^2^ = 5.96, *P* = .015). The proportions of dyslipidemia (66.7% vs 43.5%; χ^2^ = 3.09, *P* = .079), coronary artery disease (41.7% vs 17.4%; χ^2^ = 3.79, *P* = .052), and current smoking (50.0% vs 30.4%; χ^2^ = 2.20, *P* = .138) tended to be higher in the LEAD group, although these differences did not reach conventional statistical significance. Regarding laboratory indices, estimated glomerular filtration rate (eGFR) was significantly lower in the LEAD group (64.5 ± 17.7 vs 86.5 ± 17.9 mL/min/1.73 m^2^; t = −4.62, *P* < .001), suggesting more advanced renal impairment. Low-density lipoprotein cholesterol (LDL-C) levels were higher in the LEAD group (3.0 ± 0.6 vs 2.6 ± 0.6 mmol/L; t = 2.50, *P* = .016).

**Table 1:** Baseline demographic and clinical characteristics of patients with and without lower extremity arterial disease.

Variable	LEAD group (n = 36)	Non-LEAD group (n = 23)	Test statistic*	*P*-value
Age, years	70.1 ± 7.7	60.0 ± 7.7	t = 4.91	<.001
Male sex, n (%)	22 (61.1)	13 (56.5)	χ^2^ = 0.12	.726
BMI, kg/m^2^	24.8 ± 2.8	25.5 ± 3.4	t = −0.82	.414
Duration of diabetes, years	12.6 ± 4.6	7.8 ± 3.9	t = 4.29	<.001
HbA1c, %	8.4 ± 1.2	7.6 ± 0.8	t = 3.07	.003
Hypertension, n (%)	27 (75.0)	10 (43.5)	χ^2^ = 5.96	.015
Dyslipidemia, n (%)	24 (66.7)	10 (43.5)	χ^2^ = 3.09	.079
Coronary artery disease, n (%)	15 (41.7)	4 (17.4)	χ^2^ = 3.79	.052
Current smoking, n (%)	18 (50.0)	7 (30.4)	χ^2^ = 2.20	.138
eGFR, mL/min/1.73 m^2^	64.5 ± 17.7	86.5 ± 17.9	t = −4.62	<.001
LDL-C, mmol/L	3.0 ± 0.6	2.6 ± 0.6	t = 2.50	.016

BMI = body mass index, eGFR = estimated glomerular filtration rate, HbA1c = glycated hemoglobin A1c, LDL-C = low-density lipoprotein cholesterol, LEAD = lower extremity arterial disease.

### 4.2. CTA-based burden and distribution of lower extremity arterial disease

Among the 59 patients with diabetes mellitus, CTA identified lower extremity arterial stenosis or occlusion in 36 patients, corresponding to a patient level prevalence of 61.0%. At the patient level, 23 patients (39.0%) had no detectable stenosis, 6 patients (10.2%) had only mild stenosis (<50% diameter reduction), 10 patients (16.9%) had moderate stenosis (50–69% diameter reduction), and 20 patients (33.9%) had severe stenosis or occlusion (≥70% diameter reduction). Overall, clinically significant disease (defined as ≥ 50% diameter reduction) was present in 30 of 59 patients (50.8%), accounting for 83.3% (30/36) of those with any CTA-detected lesion.

For segment-level analysis, 3 major arterial segments were evaluated bilaterally in each patient (iliac–femoral, popliteal, and intrahospital), yielding a total of 177 arterial segments. CTA-detected lesions were present in 82 of 177 segments (46.3%). The proportions of segments with any stenosis or occlusion were 42.4% (25/59) in the iliac–femoral segment, 47.5% (28/59) in the popliteal segment, and 49.2% (29/59) in the intrahospital segment. With respect to severity, the proportion of segments without stenosis was 57.6%, 52.5%, and 50.8% in the iliac–femoral, popliteal, and intrahospital segments, respectively. Mild stenosis (<50%) occurred in 11.9%, 13.6%, and 10.2% of segments; moderate stenosis (50–69%) in 16.9%, 18.6%, and 16.9%; and severe stenosis or occlusion (≥70%) in 13.6%, 15.3%, and 22.0%, respectively. Although intrahospital segments showed a numerically higher proportion of severe stenosis/occlusion than the more proximal segments, the overall distribution of stenosis severity categories (none, mild, moderate, severe/occlusion) did not differ significantly among the 3 segments (χ^2^ = 2.02, *P* = .917). Among the 36 patients with CTA-detected disease, 14 patients (38.9%) had single-segment involvement and 22 patients (61.1%) had multi-segment disease (Table 2).

**Table 2:** Segment-level distribution and severity of CTA-detected lower extremity arterial lesions.

Arterial segment	Total segments, n	No stenosis, n (%)	Mild stenosis < 50%, n (%)	Moderate stenosis 50–69%, n (%)	Severe stenosis/occlusion ≥ 70%, n (%)
Iliac–femoral	59	34 (57.6)	7 (11.9)	10 (16.9)	8 (13.6)
Popliteal	59	31 (52.5)	8 (13.6)	11 (18.6)	9 (15.3)
Infrapopliteal	59	30 (50.8)	6 (10.2)	10 (16.9)	13 (22.0)

CTA = computed tomography angiography.

### 4.3. Ultrasonographic detection of lower extremity arterial disease

Clinically significant lower extremity arterial disease was defined as at least 1 arterial segment with ≥ 50% diameter stenosis or occlusion on CTA. Among the 59 patients, CTA identified clinically significant disease in 30 and no such disease in 29 cases. Using the same threshold, ultrasonography classified 34 patients as positive and 25 as negative. In comparison with CTA, ultrasonography showed a sensitivity of 90.0% (27/30) and a specificity of 75.9% (22/29), with an overall accuracy of 83.1% (49/59), a positive predictive value of 79.4% (27/34), and a negative predictive value of 88.0% (22/25). McNemar’s test indicated no significant discordance between ultrasonography and CTA at the patient level (χ^2^ = 0.90, *P* = .343). At the segment level, 177 arterial segments (iliac–femoral, popliteal, intrahospital) were evaluated. CTA demonstrated clinically significant stenosis (≥50%) in 61 segments and no significant disease in 116 segments, whereas ultrasonography classified 66 segments as positive and 111 as negative. Ultrasonography achieved a sensitivity of 88.5% (54/61) and a specificity of 89.7% (104/116), with an accuracy of 89.3% (158/177), a positive predictive value of 81.8% (54/66), and a negative predictive value of 93.7% (104/111). McNemar’s test again showed no significant discordance between ultrasonography and CTA at the segment level (χ^2^ = 0.84, *P* = .359) (Table 3).

**Table 3:** Cross-tabulation of ultrasonography versus CTA for detection of clinically significant lower extremity arterial disease at the patient and segment levels.

Level	CTA clinically significant disease (≥50%)	Ultrasonography positive (≥50%)	Ultrasonography negative	Total
Patient level	Present	27 (true positive)	3 (false negative)	30
	Absent	7 (false positive)	22 (true negative)	29
Segment level	Present	54 (true positive)	7 (false negative)	61
	Absent	12 (false positive)	104 (true negative)	116

Patient level performance: McNemar’s test χ^2^ = 0.90, *P* = .343; Segment-level performance: McNemar’s test χ^2^ = 0.84, *P* = .359.

CTA = computed tomography angiography.

To examine the impact of lesion severity on ultrasonographic detectability, segments were stratified according to CTA into mild (<50% stenosis; n = 21), moderate (50–69% stenosis; n = 31), and severe stenosis or occlusion (≥70%; n = 30). When “any lesion” on ultrasonography was defined as visible stenosis of any degree, detection rates were 52.4% (11/21) for mild, 93.5% (29/31) for moderate, and 96.7% (29/30) for severe lesions. Thus, ultrasonography was much more likely to detect moderate and severe disease than mild stenosis, and CTA-defined severity was significantly associated with ultrasonographic detectability (χ^2^ = 21.46, *P* < .001) (Table 4).

**Table 4:** Ultrasonographic detection of CTA-defined lower extremity arterial lesions stratified by lesion severity (segment level).

CTA-defined lesion severity	Segments, n	Segments with any stenosis on ultrasonography, n (%)	Segments without stenosis on ultrasonography, n (%)
Mild stenosis < 50%	21	11 (52.4)	10 (47.6)
Moderate stenosis 50–69%	31	29 (93.5)	2 (6.5)
Severe stenosis/occlusion ≥ 70%	30	29 (96.7)	1 (3.3)

Association between CTA-defined severity and ultrasonographic detection (any stenosis vs no stenosis): χ^2^ = 21.46, *P* < .001 (3 × 2 contingency table, 2 degrees of freedom).

CTA = computed tomography angiography.

### 4.4. Diagnostic performance of ultrasonography using CTA as the reference standard

The median time interval between ultrasonography and CTA was 12 days (range 1–28 days). Using CTA as the reference standard, clinically significant lower extremity arterial disease was defined as ≥ 50% diameter stenosis or occlusion in at least 1 arterial segment. At the patient level, ultrasonography correctly identified 27 of 30 patients with clinically significant disease and misclassified 3 as negative, yielding a sensitivity of 90.0% (95% CI 74.4–96.5). Among 29 patients without clinically significant disease on CTA, 22 were correctly classified as negative and 7 were overcalled as positive, corresponding to a specificity of 75.9% (95% CI 57.9–87.8). The overall accuracy was 83.1% (95% CI 71.5–90.5), with a positive predictive value (PPV) of 79.4% (95% CI 63.2–89.7) and a negative predictive value (NPV) of 88.0% (95% CI 70.0–95.8). At the segment level (177 segments), ultrasonography correctly detected 54 of 61 segments with clinically significant stenosis and misclassified 7 as negative, giving a sensitivity of 88.5% (95% CI 78.2–94.3). Among 116 non-diseased segments, 104 were correctly identified as negative and 12 were overcalled as positive, resulting in a specificity of 89.7% (95% CI 82.8–94.0). Segment-level accuracy was 89.3% (95% CI 83.8–93.0), with a PPV of 81.8% (95% CI 70.9–89.3) and an NPV of 93.7% (95% CI 87.6–96.9). When stratified by arterial territory, sensitivities for clinically significant lesions in the iliac–femoral, popliteal, and intrahospital segments were 94.4%, 90.0%, and 82.6%, respectively, and specificities were 92.7%, 89.7%, and 86.1%, respectively. Although there was a trend toward slightly lower sensitivity and specificity in the distal intrahospital segments, global χ^2^ tests showed no statistically significant differences in sensitivity (χ^2^ = 1.46, *P* = .482) or specificity (χ^2^ = 0.89, *P* = .640) among the 3 arterial segments (Table 5).

**Table 5:** Diagnostic performance of ultrasonography for clinically significant lower extremity arterial disease using CTA as the reference standard.

Level/ arterial segment	TP	FN	TN	FP	Sensitivity % (95% CI)	Specificity % (95% CI)	PPV %	NPV %	Accuracy %
Patient level (n = 59)	27	3	22	7	90.0 (74.4–96.5)	75.9 (57.9–87.8)	79.4	88.0	83.1 (71.5–90.5)
All segments (n = 177)	54	7	104	12	88.5 (78.2–94.3)	89.7 (82.8–94.0)	81.8	93.7	89.3 (83.8–93.0)
Iliac–femoral segments	17	1	38	3	94.4	92.7	85.0	97.4	93.2
Popliteal segments	18	2	35	4	90.0	89.7	81.8	94.6	89.8
Infrapopliteal segments	19	4	31	5	82.6	86.1	79.2	88.6	84.7

Global comparison of sensitivities across arterial segments (iliac–femoral vs popliteal vs infrapopliteal): χ^2^ = 1.46, *P* = .482; Global comparison of specificities across arterial segments: χ^2^ = 0.89, *P* = .640.

CI = confidence interval, CTA = computed tomography angiography, FN = false negative, FP = false positive, NPV = negative predictive value, PPV = positive predictive value, TN = true negative, TP = true positive.

## 5. Discussion

This study evaluated the diagnostic performance of lower extremity ultrasonography for the detection of clinically significant arterial disease in patients with diabetes, using CTA as the reference standard. Several main observations emerged. First, the burden of lower extremity arterial disease in this diabetic cohort was high: more than half of the patients had CTA-defined clinically significant stenosis, and almost two-thirds of those with disease had multisegmented involvement. Second, patients with LEAD were characterized by older age, longer diabetes duration, poorer glycemic control, higher prevalence of hypertension, lower eGFR, and higher LDL-C, consistent with a clustering of macrovascular risk factors. Third, ultrasonography demonstrated high sensitivity and specificity at both the patient and segment levels, with only modest reductions in performance for distal intrahospital segments and for mild lesions. These findings support the use of duplex ultrasonography as a reliable, noninvasive tool for structural vascular assessment in diabetic patients, while also highlighting areas where complementary imaging remains necessary. Compared with recent studies, our findings demonstrate comparable or slightly higher sensitivity of duplex ultrasonography in diabetic populations. Unlike prior studies that included mixed populations, our study specifically focuses on patients with diabetes, who often present with distal arterial involvement and medial calcification. This targeted analysis provides more clinically relevant evidence for this high-risk group.

The baseline characteristics observed in this study are in line with the established pathophysiology of diabetic macroangiopathy. Patients with LEAD were significantly older and had longer diabetes duration and higher HbA1c, indicating cumulative exposure to hyperglycemia and associated metabolic stress. Chronic hyperglycemia promotes endothelial dysfunction, oxidative stress, and inflammation, which contribute to accelerated atherosclerosis in large and medium-sized arteries. Coexistent hypertension and dyslipidemia further aggravate arterial wall injury and plaque formation, while renal impairment reflects systemic vascular involvement and may both result from and exacerbate atherosclerotic disease. The observed differences in eGFR and LDL-C between LEAD and non-LEAD patients reinforce the concept that LEAD is a manifestation of generalized vascular disease rather than an isolated limb condition. From an anatomic perspective, CTA revealed a substantial burden of disease across all 3 major arterial territories, with a relatively even distribution of any stenosis in iliac–femoral, popliteal, and intrahospital segments, but a numerically higher proportion of severe lesions in the intrahospital segment. This pattern is consistent with the distal predilection of diabetic atherosclerosis and the frequent involvement of crural vessels, which are critical for tissue perfusion in the foot. The high rate of multisegmented disease underscores the diffuse nature of LEAD in diabetes and explains why symptoms may be advanced at presentation, particularly in patients with concomitant neuropathy who have attenuated ischemic pain. The combination of extensive, distal disease and neuropathy is a key driver of ulceration, impaired wound healing, and limb-threatening ischemia in this population.

In this context, the diagnostic performance of ultrasonography observed in the present study is clinically relevant. At the patient level, ultrasonography achieved a sensitivity of 90.0% and specificity of 75.9% for detecting clinically significant disease (≥50% stenosis) with good overall accuracy and high negative predictive value. These metrics indicate that duplex ultrasonography is effective in ruling out clinically significant disease in most patients and provides acceptable discrimination for identifying those with substantial stenosis. At the segment level, sensitivity (88.5%) and specificity (89.7%) remained high, and performance was comparable across iliac–femoral, popliteal, and intrahospital segments, with only a tendency toward reduced sensitivity and specificity in distal segments and no statistically significant heterogeneity. The severity-stratified analysis provides additional insight into the strengths and limitations of ultrasonography. Detection rates increased markedly with lesion severity: approximately half of mildly stenotic segments (<50%) were recognized as abnormal, whereas almost all moderate (50–69%) and severe (≥70%) lesions were detected. This gradient is expected, as hemodynamic disturbances and morphological changes become more pronounced with increasing stenosis, leading to clearer alterations in flow velocity profiles, spectral broadening, and luminal narrowing on ultrasound. Conversely, mild lesions may produce subtle changes that remain below the detection threshold, especially in vessels with medial calcification or in segments with technically suboptimal acoustic windows. The high detectability of moderate and severe lesions suggests that ultrasonography is well suited for identifying the lesions of greatest clinical relevance, while the lower detection of mild disease emphasizes the need for cautious interpretation in patients with high pretest probability. These results suggest that the integration of duplex ultrasonography with functional assessments, such as ankle–brachial index or perfusion imaging, may provide a more comprehensive evaluation of disease severity. Emerging technologies, including artificial intelligence-assisted image analysis and advanced Doppler techniques, also hold promise for improving diagnostic accuracy and reducing operator dependence.

The diagnostic performance values in this study are broadly consistent with, and extend, recent evidence on duplex ultrasonography for lower extremity arterial disease. A recent analysis of duplex ultrasound in symptomatic peripheral arterial disease reported pooled sensitivities around 0.86 and specificities around 0.95 for detecting hemodynamically significant lesions, though not restricted to diabetic populations.^[10]^ In the present diabetic cohort, segment-level sensitivity (88.5%) was slightly higher and specificity (89.7%) slightly lower than these pooled values, which may reflect differences in case mix, reference standards, and the higher prevalence of medial calcification in diabetes that can complicate waveform interpretation. Several recent studies have compared duplex ultrasound with other imaging modalities in patients with diabetes. A 2025 comparative study in diabetic patients with LEAD found that duplex ultrasound, contrast-enhanced MRA, and CTA offered comparable diagnostic accuracy for detecting lower extremity arterial disease, suggesting that duplex ultrasound can serve as a robust first-line structural imaging modality when performed by trained operators.^[11]^ In a 2024 study focusing on type 2 diabetes, a combined approach using Doppler ultrasound and CTA for early detection and staging of LEAD demonstrated that Doppler ultrasound provided reliable information on lesion localization and severity, while CTA complemented it by offering panoramic visualization and detailed assessment of complex anatomy, particularly in advanced disease.^[9]^ This combined strategy aligns with the stepwise diagnostic approach observed in clinical practice and conceptually mirrors the present study design, where CTA served as the confirmatory standard.

Guidelines and contemporary reviews increasingly acknowledge duplex ultrasound as a key imaging modality in the diagnostic workup of lower extremity arterial disease. The 2024 AHA/ACC guideline on peripheral artery disease and the recent ESVS recommendations emphasize duplex ultrasound, CTA, MRA, or catheter angiography as appropriate imaging options when anatomic delineation is required, with duplex ultrasound frequently proposed as an initial noninvasive choice.^[12,13]^ In diabetic populations, the updated intersocietal IWGDF guidelines on PAD in people with diabetes and foot ulcers underline the importance of combining clinical examination, Doppler-based tests, and imaging to characterize disease burden and guide revascularization decisions.^[14]^ At the same time, recent reviews have highlighted that medial arterial calcification, which is prevalent in diabetes, can reduce the accuracy of both ankle–brachial index and duplex ultrasound in some patients and may necessitate additional imaging.^[15,16]^ This study adds to the literature by providing both patient-level and segment-level diagnostic accuracy analysis in a diabetic population, with severity-stratified evaluation, which has rarely been reported previously.

## 6. Clinical implications

The study contributes to this body of literature by providing detailed patient- and segment-level performance estimates of duplex ultrasonography in a diabetic cohort with systematically acquired CTA, quantifying performance across different arterial territories and lesion severities. The finding that ultrasonography maintains high sensitivity and specificity even in intrahospital segments is noteworthy, given reports that both ultrasound and CTA can be more challenging in distal calf vessels due to small caliber, calcification, and motion artifacts.^[7]^ These data support the use of duplex ultrasound as part of an integrated imaging pathway in diabetic patients with suspected LEAD while reinforcing the recommendation that management decisions, particularly regarding revascularization, should be informed by cross-sectional imaging when anatomy is complex or intervention is planned. Our findings support the use of duplex ultrasonography as a first-line imaging modality for screening and initial evaluation of LEAD in patients with diabetes. Given its high negative predictive value, ultrasonography may help reduce unnecessary CTA examinations, particularly in resource-limited settings.

## 7. Limitation

Several limitations should also be acknowledged. The retrospective, single-center design introduces the potential for selection bias, as only patients who underwent both ultrasonography and CTA were included, which may overrepresent individuals with a higher pretest probability of disease and limit the generalizability of the findings to broader diabetic populations, including asymptomatic individuals or those evaluated in primary care settings. The sample size, although adequate for the primary analysis, was modest, particularly for subgroup comparisons by arterial territory and for detailed exploration of specific risk factors; this may have reduced statistical power to detect small differences in diagnostic performance between segments. Operator dependence is an inherent limitation of ultrasonography; in this study, examinations were performed in a specialized vascular laboratory, and results may not be directly transferable to settings with less experience or different equipment. Interobserver variability for ultrasonography and CTA was not formally assessed, which precludes conclusions about reproducibility. Moreover, while CTA is a widely accepted anatomic reference standard, it is not infallible, especially in heavily calcified distal vessels, and comparison with digital subtraction angiography might have provided an even more stringent reference in selected cases. Finally, the study did not include functional tests such as ankle–brachial index (ABI) or toe–brachial index, nor did it evaluate long-term clinical outcomes; therefore, the prognostic implications of ultrasonography-detected lesions in this cohort remain to be defined.

In diabetic patients, extensive mural calcification introduces several potential errors. Blooming artifacts from dense calcifications can make the arterial lumen appear narrower than it truly is, leading to overestimation of stenosis severity; in extreme cases, a patent vessel may be misclassified as occluded. Conversely, beam-hardening artifacts or extremely dense concentric calcification can occasionally obscure a focal stenosis, resulting in underestimation of disease severity. These artifacts are particularly problematic in the infrapopliteal arteries, where medial calcification is highly prevalent. As such, CTA, while a widely accepted noninvasive reference standard, does not represent a perfect gold standard in this anatomical region and disease population. The imperfect reference standard must be considered when interpreting diagnostic accuracy estimates, and it reinforces the complementary role of duplex ultrasound, which can assess flow hemodynamics independently of calcification burden.

## 8. Future directions

Future prospective multicenter studies with larger sample sizes are needed to validate our findings. Additionally, incorporating quantitative ultrasound techniques and artificial intelligence-based image analysis may further improve diagnostic performance.

## 9. Conclusion

In patients with diabetes, lower extremity arterial disease was associated with older age, longer diabetes duration, suboptimal glycemic control, and an adverse cardiovascular risk profile. Ultrasonography demonstrated high diagnostic accuracy for clinically significant lesions at both the patient and segment levels. These findings support duplex ultrasonography as a reliable imaging modality.

## Author contributions

**Conceptualization:** Junxiang Zhao, Huanping Zhang, Qian Zhang, Ling Sun, Jingjing Kong, Jie Gao, Chunyan Shao.

**Data curation:** Junxiang Zhao, Huanping Zhang, Qian Zhang, Ling Sun, Jingjing Kong, Jie Gao, Chunyan Shao.

**Formal analysis:** Junxiang Zhao, Huanping Zhang, Qian Zhang, Jingjing Kong, Jie Gao, Chunyan Shao.

**Funding acquisition:** Junxiang Zhao, Ling Sun, Jie Gao, Chunyan Shao.

**Investigation:** Junxiang Zhao, Chunyan Shao.

**Writing – original draft:** Junxiang Zhao, Jingjing Kong, Chunyan Shao.

**Writing – review & editing:** Junxiang Zhao, Chunyan Shao.
